# Frailty and Cognitive Function After the Age of 40 in Adults With Moderate or Severe Congenital Heart Disease

**DOI:** 10.1016/j.cjcpc.2025.07.003

**Published:** 2025-08-05

**Authors:** Sandra Skogby, Christina Christersson, Joanna Hlebowicz, Zacharias Mandalenakis, Eva Goossens, Adrienne H. Kovacs, Liesbet Van Bulck, Koen Luyckx, Philip Moons, Camilla Sandberg, Bengt Johansson, APPROACH-IS II consortium, International Society for Adult Congenital Heart Disease

**Affiliations:** a Department of Diagnostics and Intervention, Umeå University, Umeå, Sweden; b Region Västra Götaland, Sahlgrenska University Hospital, Children’s Heart Center, Gothenburg, Sweden; c Department of Medical Sciences, Cardiology, Uppsala University, Uppsala, Sweden; d Department of Cardiology, Skåne University Hospital, Clinical Sciences, Lund University, Lund, Sweden; e Department of Molecular and Clinical Medicine, Institute of Medicine, Sahlgrenska Academy, University of Gothenburg, Gothenburg, Sweden; f Centre for Research and Innovation in Care, Department of Nursing and Midwifery, Faculty of Medicine and Health Sciences, University of Antwerp, Antwerp, Belgium; g Department of Public Health and Primary Care, KU Leuven, Leuven, Belgium; h Department of Patient Care, Antwerp University Hospital, Antwerp, Belgium; i Equilibria Psychological Health, Toronto, Ontario, Canada; j Faculty of Psychology and Educational Sciences, KU Leuven, Leuven, Belgium; k UNIBS, University of the Free State, Bloemfontein, South Africa; l Gothenburg Center for Person-Centered Care, University of Gothenburg, Gothenburg, Sweden; m Department of Paediatrics and Child Health, University of Cape Town, Cape Town, South Africa; n Department of Community Health and Rehabilitation, Umeå University, Umeå, Sweden; o Department of Public Health and Clinical Medicine, Umeå University, Umeå, Sweden

**Keywords:** heart defects, congenital, ageing

## Abstract

**Background:**

Decades of progress in care and treatment for congenital heart disease (CHD) has gradually shifted the research focus from initial survival to long-term prognosis and the ageing of adults with CHD. Knowledge about the ageing adult with CHD will guide interventions to safeguard the quality of life across the life course. The present study compares the prevalence of frailty and cognitive dysfunction between adults with CHD and a control group.

**Methods:**

Using a multicentre design, we compared adults with moderate or complex CHD aged ≥40 years, equally distributed across the age groups 40-49, 50-59, and >60 years, with age- and sex-matched controls. We assessed frailty phenotypes using the Fried method and cognitive dysfunction using the Montreal Cognitive Assessment tool.

**Results:**

In total, 156 adults with CHD (56.0 ± 10.4 years, 54.4% male) and 86 controls (55.6 ± 11.2 years, 55.8% male) were included in the study. Adults with CHD and controls did not differ in terms of mean score on the Montreal Cognitive Assessment (mean score 27.1 vs 26.9, *P* = 0.59). Similarly, there was no statistical difference in the prevalence of prefrailty/frailty between adults with CHD and controls (36.5% vs 29.0%, *P* = 0.26).

**Conclusions:**

Prevalence rates of cognitive dysfunction and frailty were similar between adults with CHD and age-matched controls. As more patients, particularly those with complex heart lesions, reach older ages, the prevalence of cognitive impairment and frailty may change.

## Résumé

**Contexte:**

Les progrès réalisés au fil des décennies dans les soins et le traitement des cardiopathies congénitales ont fait en sorte que la recherche, d’abord centrée sur la survie à court terme, s’est graduellement orientée vers l’étude du pronostic à long terme et du vieillissement des adultes atteints de cardiopathies congénitales. Les connaissances sur l’adulte vieillissant atteint de cardiopathie congénitale guideront les interventions visant à préserver la qualité de vie tout au long de la vie. La présente étude compare la prévalence de la fragilité et du dysfonctionnement cognitif entre des adultes atteints de cardiopathie congénitale et un groupe témoin.

**Méthodologie:**

Au moyen d’une étude multicentrique, nous avons comparé des adultes atteints d’une cardiopathie congénitale modérée ou complexe, âgés de 40 ans ou plus, répartis également dans les groupes d’âge de 40 à 49 ans, de 50 à 59 ans et de plus de 60 ans, à des témoins appariés selon l’âge et le sexe. Nous avons évalué les phénotypes de fragilité à l’aide de la méthode Fried et les dysfonctionnements cognitifs à l’aide du MoCA (*Montreal Cognitive Assessment*).

**Résultats:**

Au total, 156 adultes atteints de cardiopathie congénitale (56,0 ± 10,4 ans, dont 54,4 % étaient des hommes) et 86 témoins (55,6 ± 11,2 ans, dont 55,8 % étaient des hommes) ont été inclus dans l’étude. Les adultes atteints de cardiopathie congénitale et les témoins n’étaient pas différents pour ce qui est du score moyen au MoCA (score moyen de 27,1 vs 26,9, *p* = 0,59). De même, il n’y avait pas de différence statistique dans la prévalence de la préfragilité/fragilité entre les adultes atteints de cardiopathie congénitale et les témoins (36,5 % vs 29,0 %, *p* = 0,26).

**Conclusions:**

Les taux de prévalence de dysfonction cognitive et de fragilité étaient similaires chez les adultes atteints de cardiopathie congénitale et les témoins de même âge. À mesure qu’augmentera le nombre de patients atteignant un âge plus avancé, en particulier ceux qui présentent des lésions cardiaques complexes, la prévalence des troubles cognitifs et de la fragilité pourrait changer.

The congenital heart disease (CHD) population has grown immensely, and adults with CHD are now more common in our health care settings than children with CHD.^1^, ^2^, ^3^ Decades of progress in the care and treatment for CHD have allowed us to slowly shift our collective focus from initial survival to long-term prognosis and quality of life and to learn more about accelerated ageing in this population.

Adults with CHD may be exposed to residual haemodynamic abnormalities related to CHD, but also unavoidable effects of time and ageing, a combination that risks worsening CHD-related complications and health status.^4^ Indeed, both age-related morbidity and health care utilization are elevated in adults with CHD.^5^, ^6^, ^7^, ^8^, ^9^, ^10^

Cognitive decline affects quality of life and health care needs.^11^ In the general population, acquired heart disease is linked to impaired cognition and dementia.^12^, ^13^, ^14^ It would, therefore, be reasonable to hypothesize that adults with CHD are exposed to an increased risk of cognitive impairment. Patients with CHD are vulnerable to cognitive impairment, often beginning in infancy. Newborns with CHD are at risk for compromised brain development and brain injury.^12^ There are also risks associated with abnormal haemodynamics, cyanosis, and hypoperfusion.^12^ Adolescents with CHD can display difficulties in educational attainment, social functioning, or delayed progression into adulthood.^12^ Among adults with CHD, knowledge of the long-term impact of CHD on cognitive function is less available. A few studies have demonstrated that among adults, cognitive impairment seems to persist.^15^, ^16^, ^17^, ^18^ Some studies of ageing adults with CHD have reported compromised psychomotor speed and working memory,^18^ as well as an increased risk of dementia.^10^ However, other research has observed no differences in neurocognition and executive function between adults with CHD and normative data.^19^

Another important aspect related to age and ageing is frailty, which is defined as “a decline in functional reserve, resistance, and resilience of multiple organ systems, leading to an accelerated functional decline and adverse health outcomes following stressor events.”^9^^,^^20^, ^21^, ^22^ Frailty is characterized by unintentional loss of weight and muscle mass, weakness, poor endurance, and low physical activity.^23^ Frailty is often used to identify elderly individuals at risk of adverse outcomes and can be helpful in risk prediction for individuals living with chronic diseases.^24^ Frailty is generally well assessed within cardiovascular disease (CVD), and the link between CVD and frailty is considered bidirectional.^9^^,^^25^ The knowledge of frailty among adults with CHD is very limited.

The Assessment of Patterns of Patient-Reported Outcomes in Adults with Congenital Heart Disease—International Study (APPROACH-IS) recently published a substudy assessing frailty and cognitive dysfunction.^22^ The study included 814 patients from 17 different centres in 11 different countries. Overall, 38.8% of patients had some degree of cognitive dysfunction, which was associated with older age, more comorbidities, and lower country income status. The overall prevalence of frailty was 5.8%, and almost 42% were considered prefrail. Frailty was associated with older age, female sex, and comorbidities.^22^

The APPROACH-IS project contributes novel data on cognitive dysfunction in ageing adults with CHD. It has also demonstrated a high prevalence of frail and prefrail individuals in the older CHD population. However, whether frailty and cognitive dysfunction differ between adults with CHD and the general population remains unexplored. The present study evaluated cognitive function and frailty status in adults with CHD over 40 years of age and compared them with controls. Data on patients are in part reported in APPROACH-IS-II.^22^

## Methods

### Data collection

The present study is a substudy of the APPROACH-IS II study.^26^ All participants in APPROACH-IS completed a battery of patient-reported measures and patient-reported experience measures. A subset of patients also participated in functional assessments, including but not limited to frailty phenotyping and cognitive testing.^26^

Eligibility criteria for inclusion in APPROACH-IS-II included an age of 40 years or more, a CHD diagnosis of moderate or severe complexity^27^^,^^28^ made before the age of 10 years, no heart transplant, follow-up at an adult CHD clinic or inclusion in a regional/national registry, and physical, cognitive, and language abilities sufficient for self-reported questionnaires. For the present study, Swedish patients fulfilling the above criteria were included. Individuals with incomplete data or assessments were not included in the present study's analysis. The patients were recruited in approximately equal-sized subgroups: 40-49, 50-60, and 60+ years.

Control subjects were identified from the Swedish general population using a prespecified protocol to avoid selection bias. The controls were recruited based on age and sex, and participants were recruited by telephone. Both recruitment and data collection took place in 2022 at Umeå University Hospital.

### Measures

#### Sociodemographic and medical history

To collect sociodemographic variables and data on medical history, a review of medical records was conducted, combined with patient surveys.

Sociodemographic data relevant for the present study included age, sex, marital status, and educational level. Medical data included specific CHD diagnosis and New York Heart Association (NYHA) functional class as well as a history of the following: cardiac surgeries/interventions, heart failure, ventricular dysfunction, ventricular dilatation, pulmonary hypertension, hypertension, diabetes mellitus, and acquired CVD, arrhythmia, arrhythmia medications, and pacemaker or implantable cardioverter defibrillator implantation. Data on smoking status were also captured.

Ventricular function was, according to the study protocol, classed as normal, ejection fraction >50%, mild impairment, ejection fraction 40%-50%, moderate impairment 30%-40%, and severe impairment <30%. Ventricular dilation was classed according to Lang et al.^29^

Pulmonary arterial hypertension was graded according to the study protocol as no pulmonary hypertension when systolic pulmonary artery pressure ≤35 mm Hg, as mild when systolic pulmonary artery pressure 36-50 mm Hg, as moderate when systolic pulmonary artery pressure 51-60 mm Hg, as severe when systolic pulmonary artery pressure >60 mm Hg, or Eisenmenger syndrome.

The diagnosis of heart failure, past or current, was reviewed by the individual investigator as per the APPROACH-IS study protocol.^26^

To consider the impact of comorbidities on frailty, the Charlson Comorbidity Index^30^ was calculated, and patients were classified as having no comorbidities, mild comorbidities (1-3), moderate comorbidities (4-7), or severe comorbidities (8 or more).

### Frailty phenotype

A research assistant used the Fried method^22^^,^^25^ to perform frailty assessments for both patients and controls. The Fried method includes a self-reported questionnaire with items about unintentional weight loss, exhaustion, and physical activity, in addition to a walking test, and an assessment of hand grip strength by the handgrip dynamometer adjusted for sex and standing height.^26^ Frailty phenotypes were calculated based on the Fried method,^23^ which considers 5 different criteria: unintentional weight loss, exhaustion, slow gait speed, low grip strength, and low physical activity level. Each criterion is considered either positive/present or negative/not present. Participants were categorized as frail when ≥3 criteria were positive, prefrail when 1-2 were positive, and robust or nonfrail when none were positive.^23^^,^^26^

### Cognitive function

Cognitive function was assessed using the Montreal Cognitive Assessment (MoCA), a brief screening tool developed to identify individuals with cognitive impairment. The MoCA was previously evaluated in a younger CHD population.^31^ The MoCA assesses short-term memory recall, visuospatial skills, executive function, phonemic fluency, verbal abstraction, attention, concentration, working memory, language, calculation, and orientation. It is a 1-page, 30-point test that takes approximately 10 minutes to administer by a research assistant.^26^^,^^32^

To determine cognitive impairment, total MoCA scores were calculated. Individuals with ≤12 years of formal education were granted an additional point. A cutoff score of 26 points was used, meaning that scores ≤25 were considered to indicate cognitive dysfunction.^32^

### Statistical analysis

Results are reported as absolute number and percentages for categorical variables and mean ± standard deviation or median with interquartile range for continuous variables. Differences between patients and controls were assessed with the χ^2^ test (ratios), the Fisher exact test (ratios with a small, expected number in at least 1 cell), the Student *t* test (means), or the Mann-Whitney *U* test (ranks). Associations with the frailty phenotype and cognitive dysfunction were assessed using logistic regression analysis. For *P* values <0.05, the null hypothesis was rejected.

IBM SPSS Statistics for Windows, version 29 (IBM Corp, Armonk, NY) was used for statistical analysis.

### Ethical considerations

Written informed consent was obtained from all study participants. The study was approved by the Regional Ethics Review Board (Swedish registration number Dnr 2019-06247) and performed in accordance with the Declaration of Helsinki (2013).^33^

## Results

After excluding 13 cases and 2 controls due to incomplete data or assessments, 156 adults with CHD and 86 controls were included in the analysis.

### Sample characteristics

The mean age was 56.0 ± 10.4 years, and 54.5% were male. Most were nonsmokers, married or living with a partner, and had a high school education or above. Coarctation of the aorta was the most common CHD diagnosis, representing 34.6%, followed by repaired tetralogy of Fallot (16.7%) and congenital aortic valve disease (16.0%). Severe CHD complexity was present in 12.8%, and 22.4% had NYHA class 2 or above. The majority had no arrhythmia, pulmonary hypertension, heart failure, ventricular dysfunction, or dilation (Tables 1 and 2).

**Table 1:** ACHD characteristics (n = 156)

Characteristic	Value, n (%)	Percentage with at least 1 surgical intervention∗
CHD diagnosis		
Complex CHD		
TGA (classic or d-TGA with atrial switch procedure, ccTGA)	12 (7.7)	75†
Fontan physiology	3 (1.9)	100
Pulmonary atresia (all forms)	3 (1.9)	100
Single ventricle	1 (0.6)	100^1^
Double-outlet ventricle	1 (0.6)	100
Moderate CHD		
Coarctation of the aorta	54 (34.6)	94.5‡
Repaired tetralogy of Fallot	26 (16.7)	100
Isolated congenital aortic valve disease (aortic stenosis and regurgitation)	25 (16.0)	80
AVSD (partial or complete, including primum ASD)	8 (5.1)	100^a^
Ebstein anomaly (only moderate or severe Ebstein included)	6 (3.8)	40^a^
Isolated pulmonary valve stenosis (moderate or great)	6 (3.8)	100
Isolated pulmonary valve regurgitation (moderate or great)	2 (1.3)	100
VSD (with associated mild abnormality and/or moderate or great shunt—Qp:Qs >1.5)	2 (1.3)	50§
Sinus venosus defect	2 (1.2)	100
Infundibular right ventricular outflow obstruction	1 (0.6)	100
Moderate and large persistently patent ductus arteriosus	1 (0.6)	100^2^
Sub- and supravalvular aortic stenosis (excluding HCM)	2 (1.2)	50
Other defects of moderate complexity	1 (0.6)	0
CHD complexity		
Severe complexity	20 (12.8)	
Moderate complexity	136 (87.2)	
Surgical interventions∗	b	
No surgical intervention	17 (11)	
1 surgical intervention	66 (42.8)	
2 surgical interventions	45 (29.2)	
3 surgical interventions	20 (12.9)	
>3 surgical interventions	6 (3.9)	
Interventional catheterizations∗		
No interventional catheterization	131 (84)	
1 interventional catheterization	19 (12.2)	
2 interventional catheterizations	5 (3.2)	
3 interventional catheterizations	1 (0.6)	
NYHA class		
Class I	121 (77.6)	
Class II	29 (18.6)	
Class III	6 (3.8)	
Arrhythmia	b	
No arrhythmia	99 (63.5)	
Yes, but not require treatment	9 (5.8)	
Yes, stable on treatment	40 (25.6)	
Yes, but refractory arrhythmia	6 (3.8)	
Arrhythmia medication	34 (21.8)	
ICD	6 (3.8)	
Pacemaker	21 (13.5)	
Ventricular dysfunction	c	
Mild	36 (23.1)	
Moderate/severe	13 (8.3)	
Ventricular dilatation	e	
Mild	35 (23.1)	
Moderate/severe	19 (12.5)	
Pulmonary hypertension	e	
Mild	5 (3.3)	
Moderate/severe	2 (1.3)	
Severe	2 (1.3)	
Heart failure	c	
Diagnosis of heart failure	26 (15.0)	

a = Data missing for 1 participant.

b = Data missing for 2 participants.

c = Data missing for 3 participants.

e = Data missing for 5 participants. 1. No palliative shunt. 2. No cyanosis, no Eisenmenger.

The definition of moderate/great shunt was >Qp/Qs >1.5.

ACHD, adult congenital heart disease; ASD, atrial septal defect; AVSD, atrioventricular septal defect; ccTGA, congenitally corrected transposition of the great arteries; CHD, congenital heart disease; d-TGA, d-transposition of the great arteries; HCM, hypertrophic cardiomyopathy; ICD, implantable cardioverter defibrillator; NYHA, New York Heart Association Functional Class; TGA, transposition of the great arteries; VSD, ventricular septal defect.

∗ Data on “surgical interventions” and “interventional catheterisations” are limited to whether cardiac intervention was performed or not and do not include information on the specific intervention.

† Three patients without surgical intervention classified as ccTGA.

‡ For patients with coarctation of the aorta, the percentage with surgery could be either surgery or interventional catheterization.

§ One patient had surgical repair of a VSD in combination with a mild heart lesion and therefore classified as moderate CHD.

**Table 2:** Demographics

Demographics	ACHD (n = 156)	Controls (n = 86)	*P* value
Sex			0.89
Male	85 (54.5)	48 (55.8)	
Female	71 (45.5)	38 (44.2)	
Age			0.92
Mean ± SD	56.0 ± 10.4	56.0 ± 11.2	
Minimum-maximum	40-78	41-79	
Height (m)			0.42
Mean ± SD	1.7 ± 0.1	1.7 ± 0.1	
Weight (kg)			0.42
Mean ± SD	81.4 ± 17.6	83.2 ± 15.0	
Smoking	d		0.13
Never smoker	116 (74.4)	56 (65.1)	
Previous smoker	30 (19.2)	27 (31.4)	
Active smoker	6 (3.8)	3 (3.5)	
Disease burden			
Diagnosis of diabetes	10 (6.4)^d^	5 (5.8)^a^	1.00
Diagnosis of hypertension	57 (36.5)^d^	22 (25.6)^b^	0.08
Previous stroke, TIA, myocardial infarction, or PCI	12 (7.7)^g^	5 (5.8)^a^	0.61
Charlson Comorbidity Index			0.001
No comorbidities	144 (92.3)	78 (90.7)	
Mild comorbidities	33 (21.1)	5 (5.8)	
Moderate comorbidities	9 (5.7)	2 (2.3)	
Severe comorbidities	0	1 (1.1)	
Marital status		a	0.015
Never married	17 (10.9)	2 (2.3)	
Married/living with a partner	125 (80.1)	70 (81.4)	
Divorced/widowed	14 (9.0)	11 (12.8)	
Other	0	2 (2.3)	
Educational level	b	b	0.25
No complete high school education	20 (12.8)	6 (7.0)	
Complete high school education	74 (47.4)	39 (45.3)	
Bachelor’s degree	40 (25.6)	21 (24.4)	
Master’s degree	20 (12.8)	18 (20.9)	

Continuous variables were analysed with the Man-Whitney *U* test.

Categorical variables were analysed with the χ^2^ test, the Fisher exact test, or the Fisher-Freeman-Halton exact test.

a = missing data for 1 participant, b = missing data for 2 participants, d = missing data for 4 participants, g = missing data for 7 participants.

ACHD, adult congenital heart disease; PCI, percutaneous coronary intervention; SD, standard deviation; TIA, transient ischemic attack.

### Cognitive function

**Figure 1:**
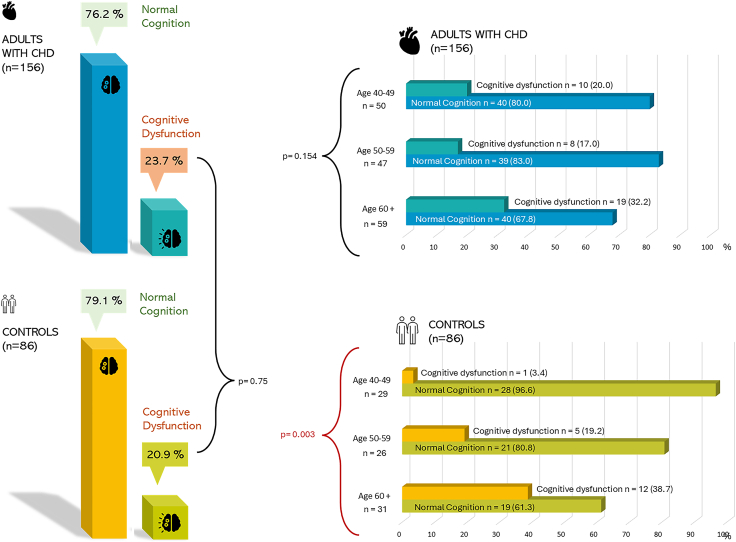
An overview of normal cognition and cognitive dysfunction proportions among cases and controls. The figure also displays proportions of normal cognition and cognitive dysfunction across the 3 age groups 40-49, 50-59, and 60 plus across both cases and controls. Differences across cases and controls as a whole and across the age groups in terms of cognitive dysfunction are reported. CHD, congenital heart disease.

Adults with CHD and controls did not differ in terms of mean score on the MoCA (mean score 27.1 vs 26.9, *P* = 0.59). Neither did the proportions of participants with cognitive dysfunction (ie, ≥26) differ between adults with CHD and controls (23.7% vs 20.9%; *P* = 0.64) (Fig. 1).

Among controls, cognitive function differed across age groups; however, among adults with CHD, it did not (Fig. 1). Complex CHD was less common in ageing cohorts as follows: 24% among patients aged 40-49 years, 10.6% among patients aged 50-59 years, and 5.1% among patients aged ≥60 years (*P* = 0.010). Although 20.0% of patients aged 40-49 years had cognitive dysfunction and 32.2% of patients aged ≥60 years had cognitive dysfunction, the difference in the prevalence of cognitive dysfunction between the 3 age categories was not statistically significant (*P* = 0.154) (Fig. 1). Among controls, however, rates of cognitive dysfunction were higher across older age groups (*P* = 0.003) (Fig. 1).

As a subgroup analysis, cognitive dysfunction was compared between patients and controls within each of the 3 age categories. The rates of cognitive dysfunction did not differ between patients and controls in the older 2 categories, although the prevalence was higher among patients than controls aged 40-49 years (20.0% vs 3.4%; *P* = 0.048).

Among controls, cognitive dysfunction was associated with increasing age (odds ratio [OR]: 1.09, 95% confidence interval [CI]: 1.03-1.15). Among adults with CHD, the point estimate indicates a similar association between cognitive dysfunction and age (OR: 1.03, 95% CI: 0.99-1.07). Among adults with CHD, cognitive dysfunction was further associated with comorbidities (*P* = 0.021); however, it was not associated with sex, CHD complexity, or NYHA class.

### Frailty

**Figure 2:**
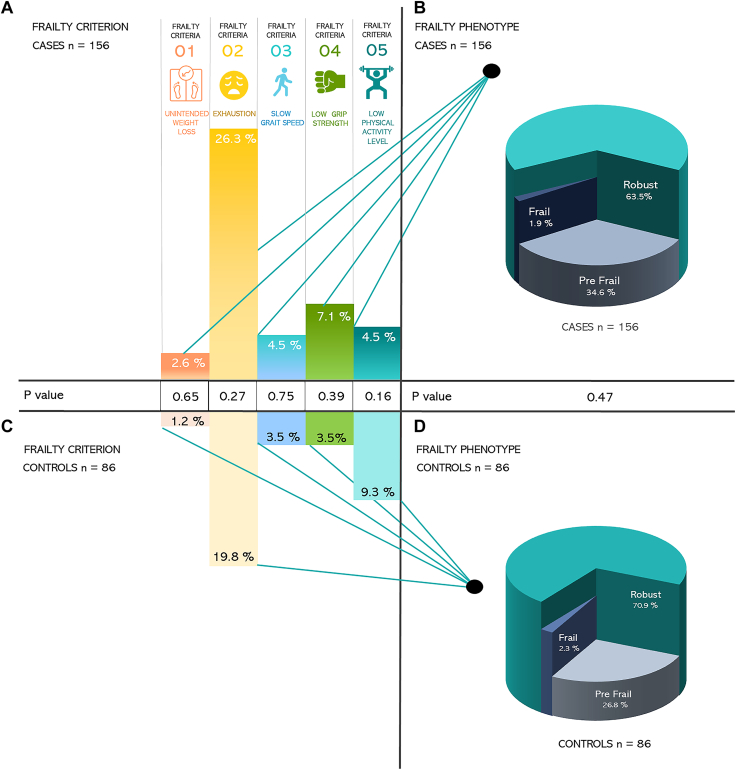
An overview of the prevalence of positive frailty criteria among cases vs controls in panel (**A**) and panel (**C**). The figure also displays proportions of frailty phenotypes across cases and controls in panel (**B**) and panel (**D**). Differences across cases and controls in terms of positive frailty criteria and frailty phenotypes are also reported.

The most common frailty criterion was exhaustion, reported by 26.3% of patients and 19.8% of controls (Fig. 2). There were no differences in the prevalence of any of the frailty criteria between patients and controls (Fig. 2). Similarly, there were no differences between patients and controls in the prevalence of frailty phenotype (3 levels) (Fig. 2) or when comparing frail or prefrail status between patients and controls (n = 57 [36%] vs n = 25 [29%], *P* = 0.26).

Among adults with CHD, the presence of frailty or prefrailty was associated with NYHA class (OR: 2.59, 95% CI: 1.20-5.59) and comorbidities (OR: 2.5, 95% CI: 1.24-5.10). However, being frail or prefrail was not associated with age, sex, CHD complexity, hypertension, arrhythmia, or acquired heart disease.

## Discussion

We investigated frailty and cognition among adults with CHD aged ≥40 years, comparing them with controls.

The rate of cognitive dysfunction did not differ between patients and controls. Furthermore, there were no differences between patients and controls in any of the individual frailty criteria, or in the overall prevalence of frailty or prefrailty.

Our results strengthen the notion that ageing in adults with CHD, on a group level, is not associated with either cognitive dysfunction or frailty. However, there are indications that cognitive dysfunction may be a concern in the younger age group (40-50 years), possibly reflecting the fact that more patients with severe CHD are reaching older ages.

### Cognition

The finding of similar overall rates of cognitive dysfunction between patients and controls was unexpected, given the patients' elevated risk of neurodevelopmental deficits and disabilities in infancy and childhood, as well as their exposure to adverse haemodynamics throughout their lifespan.^12^ Furthermore, early-onset dementia has been reported among adults with CHD.^10^^,^^12^ In addition, the main APPROACH-IS-II study showed a higher proportion of cognitive dysfunction than in this substudy^22^—38.8% vs 23.7% reported here. This difference was confirmed with a *post hoc* χ^2^ test (*P* < 0.001). In this context, it is worth noting that the main APPROACH-IS-II study represents a much broader population, recruited from 11 countries, thereby reflecting a diverse range of health care systems.

Among individuals aged 40-49 years, the prevalence of cognitive dysfunction in adults with CHD was almost 6 times that of the prevalence in controls. However, these results should be interpreted with caution, given the study's limited power for subgroup analysis. However, some reflection on these results still seems appropriate. The proportion of complex CHD was higher among younger cases, which likely explains the higher rate of cognitive dysfunction in this age group. Indeed, cognitive impairment has been reported to be more common in complex CHD.^12^ A higher prevalence of complex CHD among younger cases is expected, given the recent increase in survival rates. As survival continues to improve, the proportion of older adults with severe CHD will also increase over time. More speculatively, it could be that the older age group represents survivors with more favourable outcomes of their CHD and thus a lower prevalence of cognitive dysfunction.

In the previously published APPROACH-IS-II study, cognitive dysfunction varied by age, comorbidities, and country income status.^22^ However, we did not observe such differences by age or comorbidities in our study. The present study is limited to Sweden, a high-income country with well-structured care for individuals with CHD, which may contribute to the differences observed when compared with the larger APPROACH study. In addition, it is important to consider the differences in sample size and statistical power between the present study and the main APPROACH study, which limit the ability to perform subgroup analyses in our study.

Previous studies have also used the MoCA to assess cognitive function in patients with CHD. In a study by Pike et al.^31^ examining youth with CHD, the prevalence of cognitive dysfunction was significantly higher among patients (69%) compared with controls (13%) in a cohort where 59% had complex CHD. This finding is not unexpected, as younger individuals with CHD more often have complex cardiac lesions, which are associated with cognitive impairment, whereas cognitive dysfunction is rare in young, healthy controls.

In contrast, our present cohort of patients with CHD is older and exhibited better cognitive performance than that reported by Pike et al.^31^ This difference may be attributed to variations in CHD complexity, as 59% of the Pike et al.^31^ cohort had complex CHD, whereas in our study, only 24% of the youngest subgroup had complex CHD.

### Frailty

Regarding frailty, our sample of Swedish adults with CHD exhibited a lower prevalence of frailty (1.9%) than the full APPROACH-IS-II dataset (5.8%).^22^ In addition, the prevalence of frailty among our control group differs from previous studies examining the general population.^29^, ^30^, ^31^ For instance, Gordon et al.^34^ reported that approximately 40% of individuals were either frail or prefrail, whereas in our study, this figure was 29%. These differences may be attributed to geographical and contextual factors, as well as variations in inclusion criteria across studies.

The most met frailty criterion among our adults with CHD was exhaustion, which aligns with findings from the larger APPROACH-IS-II study.^22^ However, in contrast to the main APPROACH-IS-II study, our substudy did not find an association between frailty and age, sex, comorbidities, or physiological state.

### Methodological considerations

A major strength of this study is the comparison between adults with CHD and a control group, as well as the use of the Fried frailty phenotype and MoCA tools, both of which are validated and widely applied in various settings.^23^^,^^32^

However, some limitations should be considered when interpreting our findings. First, the relatively small sample size may limit statistical power in subgroup analyses. Second, as previously mentioned, there is a risk of under-representation of complex CHD among older patients. This reflects the general epidemiology, as patients with the most advanced interventions have not yet reached older ages. In addition, a survival bias may be present in the older subgroup, meaning that individuals with more complex CHD may have died earlier. Moreover, certain CHD lesions that have only recently begun to reach adulthood have not yet been observed in older age groups. This is an expected consequence of the evolving epidemiology of CHD.

Third, the prevalence of arterial hypertension increases with age and has been linked to cognitive dysfunction. In individuals with coarctation of the aorta, hypertension is a common comorbidity. However, data regarding the prevalence of hypertension in either case or control groups are lacking in our study. Furthermore, in the context of CHD, numerous additional factors may contribute to cognitive impairment, making it challenging to isolate the specific effects of arterial hypertension on cognitive outcomes.

Fourth, the study focuses on a selected group of adult patients with CHD, excluding those with severe cognitive impairment or associated syndromes that precluded participation per study protocol. Fifth, given that both frailty and cognitive dysfunction have been shown to vary geographically,^22^ the findings of this study might be more applicable to high-income countries or certain types of health care system. Consequently, the prevalence of frailty and cognitive dysfunction reported here should not be assumed to be generalizable to other populations or contexts.

## Conclusions

Cognitive dysfunction and frailty did not differ between adults with CHD and controls living in Sweden. The prevalence of cognitive function and frailty may change in the future when more patients, especially those with complex heart lesions, reach a higher age.
